# Hépatites Delta en Afrique: particularités épidémiologiques et cliniques

**DOI:** 10.48327/mtsi.v3i4.2023.430

**Published:** 2023-10-03

**Authors:** Françoise LUNEL FABIANI, Ahmed EL BARA, Cheikh Tijani HAMED, Hélène LE GUILLOU GUILLEMETTE

**Affiliations:** 1 Service de virologie, Centre hospitalier universitaire d'Angers, Angers, France; 2 Université d'Angers, Laboratoire HIFIH (Hémodynamique, interaction fibrose et invasivité tumorales hépatiques), EA 3859, Angers, France; 3 INRSP (Institut national de recherche en santé publique), Nouakchott, Mauritanie; 4 INHV (Institut national d'hép ato-virologie), Nouakchott, Mauritanie

**Keywords:** VIH, VHB, VHD, Sévérité des maladies hépatiques, Afrique, Mauritanie, HIV, HBV, HDV, Liver disease severity, Africa, Mauritania

## Abstract

En 2022, l'Organisation mondiale de la santé (OMS) estimait que les infections par le virus de l'hépatite B (VHB) avaient causé 1,5 millions de décès, imputables pour la plupart aux complications des infections chroniques, cirrhose et carcinome hépatocellulaire (CHC). Malgré la disponibilité d'un vaccin, 296 millions de personnes étaient chroniquement infectées en 2019. L'Asie et l'Afrique sont les continents les plus impactés par cette infection, avec pour l'Afrique entière environ 100 millions de sujets infectés.

Le virus de l'hépatite Delta ou D (VHD), qui est un virus « satellite » du VHB, est souvent méconnu et son diagnostic reste négligé. Il est pourtant associé à des formes fulminantes aiguës et des formes chroniques d'hépatite entraînant une évolution plus rapide vers la cirrhose et le CHC que lors de la mono-infection par le VHB. La recherche sur ces deux virus VHB et VHD a beaucoup progressé ces dernières années, et de nouveaux traitements sont actuellement en développement.

Chez les personnes vivant avec le virus de l'immunodéficience humaine (PvVIH), les maladies hépatiques représentent une cause majeure de morbidité et de mortalité. Du fait des modes de transmission communs, les doubles ou triples infections VIH/VHB ou VIH/VHB/VHD sont relativement fréquentes, en particulier dans les régions d'endémicité du VHB comme l'Afrique. Cependant, si aujourd'hui la plupart des patients co-infectés bénéficient d'un traitement efficace à la fois contre le VIH et le VHB, celui-ci n'est pas actif sur le VHD. En Afrique, les hépatites B et D ont déjà fait l'objet de plusieurs travaux. Cependant, la fréquence et les conséquences cliniques de ces co-infections n'ont été que peu étudiées en population générale et chez les PvVIH.

Cette mise au point cherche à actualiser les données épidémio-cliniques et les perspectives thérapeutiques des co-infections par le VHD ou triples infections (VIH/VHB/VHD) en Afrique.

## Abstract (en)

In 2022, the World Health Organization (WHO) estimated that hepatitis B virus (HBV) infections caused 1.5 million deaths, mostly attributable to complications from chronic infections, cirrhosis and hepatocellular carcinoma (HCC). Despite the availability of a vaccine, 296 million people were chronically infected in 2019. Asia and Africa are the continents most affected by this infection, with around 100 million people infected in Africa as a whole.

Hepatitis Delta or D virus (HDV), which is a “satellite” virus of HBV, is often misunderstood and its diagnosis remains neglected. However, it is associated with acute fulminant forms and chronic forms of hepatitis leading to a more rapid evolution towards cirrhosis and HCC than during HBV mono-infection. Research on these two viruses HBV and HDV has progressed a lot in recent years, and new treatments are currently in development.

In people living with the human immunodeficiency virus (PlHIV), liver disease is a major cause of morbidity and mortality. Due to common modes of transmission, dual or triple HIV/HBV or HIV/HBV/HDV infections are relatively common, particularly in HBV endemic regions such as Africa. However, while today most co-infected patients benefit from effective treatment against both HIV and HBV, the latter is not active against HDV. In Africa, hepatitis B and D have already been the subject of several studies. However, the frequency and clinical consequences of these co-infections have been little studied in the general population and in PlHIV.

This review seeks to update the epidemiological and clinical data and the therapeutic perspectives of HDV co-infections or triple infections (HIV-HBV-HDV) in Africa.

## Introduction

Selon l'OMS, en 2019, 316 millions [43] de personnes étaient chroniquement infectées par le virus de l'hépatite B (VHB) dans le monde avec un risque élevé de développer une maladie hépatique sévère. En Afrique, toujours selon l'OMS, on dénombre plus de 100 millions de porteurs du VHB [56, 84]. L'infection chronique par le VHB représente donc, encore aujourd'hui, un problème de santé majeur avec, chez les personnes porteuses chroniques de l'antigène HBs (AgHBs) à travers le monde, plus de 820 000 morts chaque année des complications liées à l'infection VHB. Le carcinome hépatocellulaire (CHC) est la troisième cause de mortalité par cancer, la plupart des cas résultant d'une infection chronique par le VHB. Il est très fréquent en Afrique [26].

Les traitements actuels permettent de contrôler la réplication mais ne permettent pas d’éliminer le virus du fait de sa persistance sous forme d'ADN circulaire covalent clos (ADNccc) dans le noyau des hépatocytes infectés [36]. Selon des estimations en 2020, seulement 30,4 millions des personnes infectées connaissaient leur statut et seulement 6,6 millions bénéficiaient d'un traitement antiviral [84]. Seul un petit nombre de pays africains disposent de plans d'action nationaux pour lutter contre les hépatites virales. De nombreux obstacles empêchent le développement de stratégies nationales visant à améliorer la prévention, la prise en charge et le traitement des hépatites virales [43].

Le virus de l'hépatite Delta (VHD) fait partie des pathologies les plus négligées, en particulier dans les pays à ressources limitées. En effet, son diagnostic est rarement fait, en raison de la méconnaissance de cette infection et du prix des réactifs. Il est pourtant associé à des formes graves, aiguës fulminantes et chroniques entraînant une évolution plus rapide vers la cirrhose et le CHC que lors de la mono-infection par le VHB [26]. Avec la découverte du récepteur cellulaire du VHB et une meilleure compréhension de la multiplication virale, d’énormes progrès ont été faits et de nouveaux traitements sont en développement [62].

Dans le monde, plus de 38,4 millions de personnes sont infectées par le virus de l'immunodéficience humaine (VIH) et les deux tiers vivent en Afrique subsaharienne [85].

Le VIH, le VHB et le VHD partagent des voies de transmission similaires et des zones d'endémicité communes [120]. Chez les personnes vivant avec le VIH (PvVIH), les maladies hépatiques représentent une cause majeure de morbidité et de mortalité, notamment depuis la démocratisation des thérapies antirétrovirales (ARV) qui ont permis l'allongement de leur espérance de vie [106]. Les statistiques estiment que 10% des PvVIH sont co-infectées par le virus de l'hépatite B [42]. De plus, environ 14% des patients atteints d'hépatite B seraient également infectés par le VHD avec des prévalences variables en fonction du pays [40].

Nous présentons ci-après un état des lieux sur ces doubles ou triples infections en Afrique, en commençant par un rappel rapide sur les infections par le VHB en Afrique, qui nous a paru indispensable, en raison de l’étroit lien entre ces 2 infections.

## L'HEPATITE B

### Aspects virologiques

Le VHB est un virus enveloppé à ADN circulaire, partiellement double brin. L'enveloppe entoure la nucléocapside icosaédrique qui elle-même entoure le génome viral qui se présente sous la forme d'une molécule d'ADN de 3200 paires de bases. Le virus dispose de quatre cadres de lecture ouverts (ORF/Open Reading Frame) nommés S, C, P et X. Les gènes S, pré-S1 et pré-S2 codent pour les protéines d'enveloppe. Le gène C code pour la protéine de capside et la région pré-C code pour l'AgHBe. La région P code pour l'ADN polymérase virale qui possède à la fois des activités de transcriptase inverse, d'ADN polymérase ADN-dépendante et de RNase H. La région X code pour le polypeptide X, protéine transactivatrice possédant un potentiel oncogène [52, 104, 105]. Les niveaux élevés de production virale confèrent au VHB un fort potentiel de variabilité génétique [92]. Il en résulte une variabilité inter-individuelle du VHB, avec 10 génotypes (A à J) et plusieurs sous-génotypes identifiés [5, 129]. Les génotypes prédominants en Afrique sont les génotypes A, D et E [119].

Il existe également une variabilité phénotypique du VHB. Des mutants peuvent être sélectionnés sous la pression immunologique ou médicamenteuse [112]. On dénombre principalement:

- des mutants dans l'enveloppe, responsables d'un certain nombre d’échecs de la vaccination anti-VHB;
- des mutants dans la polymérase, qui sont responsables de résistance aux antiviraux, comme la lamivudine;
- et enfin, des mutants du promoteur basal du core (BCP) et du gène Pré-C qui se caractérisent par des hépatites chroniques AgHBe négatives et, pour les mutants dans le BCP, une diminution de la production de l'AgHBe. Ces mutants sont associés, pour certains auteurs, à une plus grande sévérité de la maladie hépatique [32].

### Aspects épidémiologiques

L'OMS distingue 3 zones d'endémicité [87]:

- une zone de faible endémicité caractérisée par un taux de positivité de l'AgHBs inférieure à 2% correspondant aux pays d'Europe de l'Ouest et d'Amérique du Nord [32];
- une zone d'endémicité intermédiaire, où la prévalence de l'AgHBs oscille entre 2 et 8%, englobant l'Asie du Sud-Est et le bassin méditerranéen oriental;
- une zone d'endémicité élevée supérieure ou égale à 8% regroupant l'Afrique, avec 100 millions environ de sujets infectés, et les régions du Pacifique occidental avec également près de 100 millions de porteurs chroniques [16, 87, 91, 101].

**Figure 1:**
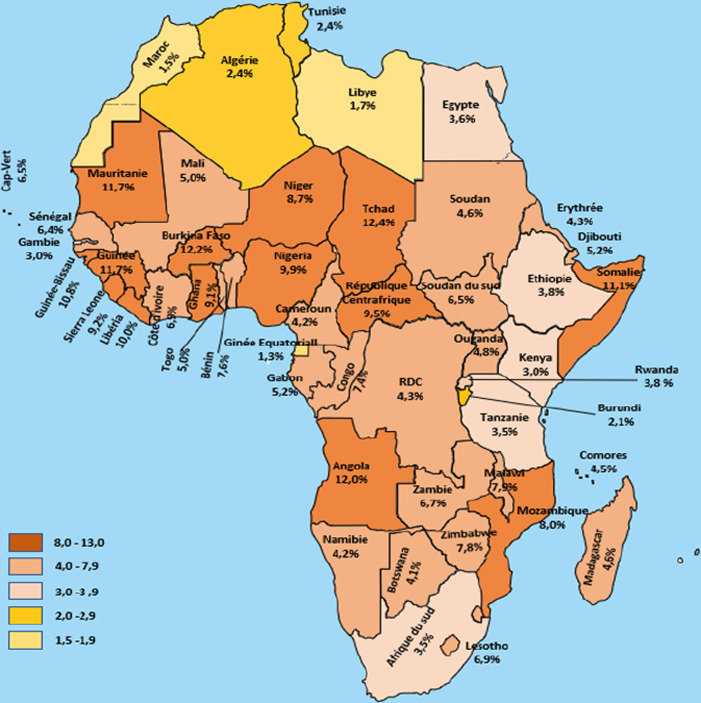
Prévalence de l'hépatite B en Afrique, d'après GBD 2019 Hepatitis B collaborators [43], http://ghdx.healthdata.org/ Prevalence of hepatitis B in Africa, from GBD 2019 Hepatitis B collaborators [43], http://ghdx.healthdata.org/

L'OMS estime l'incidence de l'infection par le VHB à plus de 1,5 million de cas par an dans le monde [43, 84]. On estime que 56 à 98 *%* des Africains ont été en contact avec le virus de l'hépatite B et 70% des cas d'hépatite B dans le monde sont concentrés en Afrique [108]. L’épidémiologie de l'infection par le VHB en Afrique est difficile à apprécier car la plupart des études n'ont recherché que l'AgHBs, et non les marqueurs indirects d'infection ancienne en particulier les Ac anti-HBc [14]. L'Afrique du Nord est considérée comme une zone d'endémicité intermédiaire avec une prévalence de 2 à 7%, tandis que l'Afrique subsaharienne est une zone de haute endémicité avec une prévalence comprise entre 8 et 18% [43, 64] (Fig. 1). Le Maghreb semble donc peu touché avec des taux de prévalence de l'ordre de 1,09% au Maroc et 2,89% en Algérie [101]. En revanche, l'Afrique subsaharienne a des taux de prévalence très importants rapportés en Guinée (10,8%) [12], au Liberia (10%), au Niger (8,7%), au Mali (5%) [43], au Nigeria (9,9%) [43], au Sénégal (6,4%) [43] et au Cameroun (4 à 20%) [31]. En Mauritanie, des études montrent une prévalence allant de 10,7% de l'AgHBs chez les femmes enceintes, 15% chez les donneurs de sang jusqu’à 17,15% chez les patients hospitalisés [74, 78, 79].

Dans ces régions, la proportion des infections chroniques en fait la principale cause de cirrhose et de carcinome hépatocellulaire avec une mortalité élevée et des conséquences socio-économiques importantes [34, 88].

Sur le plan vaccinal, selon l'OMS, en 2021, la couverture de la vaccination systématique des enfants contre l'hépatite B était estimée à 72 *%* dans la région, ce qui est insuffisant. La vaccination à la naissance est administrée seulement dans 14 pays africains [14].

### Aspects cliniques, histoire naturelle et évaluation de la maladie hépatique

L'infection aiguë par le VHB va entraîner une hépatite aiguë, fulminante dans 1 *%* des cas environ [116]. Dans 5 à 10% des cas, chez l'adulte, l'infection devient chronique. Elle le devient aussi chez 30% des enfants infectés avant 5 ans et jusqu’à 90% chez le nourrisson contaminé par transmission verticale [35]. L’évolution naturelle de l'infection chronique par le VHB est caractérisée par 5 phases basées sur la présence ou non de l'AgHBe, les taux de l'ADN du VHB et des transaminases [35]. On distingue ainsi:

1. l'infection chronique AgHBe positive;
2. l'hépatite chronique AgHBe positive;
3. l'infection chronique AgHBe négative;
4. l'hépatite chronique AgHBe négative;
5. l'hépatite résolutive avec perte de l'AgHBs.

L’évaluation de la sévérité de l'atteinte hépatique est essentielle pour établir le stade de l'infection chronique et décider ou non de l'initiation d'un traitement. Elle reposait autrefois sur la ponction biopsie hépatique (PBH). Aujourd'hui des marqueurs non invasifs sont utilisés. Ces tests ou méthodes ont été validés chez les adultes atteints d'hépatite B chronique (HBC). Ils sont utilisés essentiellement pour exclure une fibrose avancée.

L’« aspartate aminotransferase to platelet » ratio index (APRI) correspond au rapport entre les taux des plaquettes et des ASAT. Le « *fibrosis-4 index* » (FIB-4) est basé sur un score, calculé à partir des taux d'ASAT, d'ALAT, des plaquettes et de l’âge [109]. Les scores FIB-4 et APRI sont peu coûteux mais comportent des biais lorsqu'il existe d'autres causes d’élévation des transaminases et de thrombopénie; une variabilité forte en fonction de l’âge est aussi notée pour le FIB-4.

Les marqueurs commerciaux sanguins tels que le FibroTest^®^ [93] et le Fibromètre^®^ [17], ou morphométriques tels que l’élastographie impulsionnelle (FibroScan^®^)pourraient être utilisés préférentiellement; leur fiabilité est meilleure, mais ils sont onéreux et pas toujours aisément accessibles. En l'absence de signes cliniques de cirrhose, l’évaluation de la fibrose se fait idéalement sur la base de deux tests sanguins (APRI et FIB-4) ou le FibroScan^®^ et un test sanguin [109].

Des recommandations pour le traitement des hépatites B ont été faites par les sociétés savantes (AFEF, EASL, AASLD, APASL) et sont reprises dans une revue récente du *Lancet* et par l'OMS [56]. En résumé, en Afrique, les analogues nucléotidiques utilisés dans les infections à VIH sont privilégiés et les indications reposent sur la présence d'une fibrose (marqueurs indirects) et/ou d'une augmentation prolongée des transaminases (correspondant aux phases 2 et 4 de l'infection, citées plus haut) [35].

## L'HÉPATITE DELTA

### Aspects virologiques

L'antigène Delta a été identifié chez des patients atteints d'une infection chronique par le VHB par Rizetto en 1977 [95]. L’équipe de Wang a réussi à cloner et séquencer le génome du VHD dans les années 1980 [121]. La particule virale Delta est sphérique, d'un diamètre de 36 nm, l'enveloppe est constituée par les protéines de l'enveloppe du VHB. Elle entoure une nucléocapside qui contient l'antigène Delta et l'ARN génomique [65]. Le génome est constitué d'un brin d'ARN monocaténaire de polarité négative composé d'environ 1680 nucléotides [66]. L'antigène Delta est composé de deux isoformes S pour *small* de 24 kDa et L pour *large* de 27 kDa qui jouent un rôle très important dans le cycle viral [3, 114].

Le génome du VHD possède aussi un cadre de lecture ouvert qui code pour les 2 isoformes de la protéine Delta. La réplication du génome du VHD s'effectue dans le noyau par un mécanisme de cercle roulant [20, 113]. Le VHD a également une diversité génétique importante, qui a conduit à une classification en 8 génotypes. Le génotype 1 est le plus fréquent et est trouvé sur tous les continents, le plus souvent associé au génotype D du VHB. Les génotypes 5 à 8 sont présents en Afrique centrale et de l'Ouest avec le génotype 1 identifié notamment au Cameroun, Gabon, Nigeria, Ghana et en Mauritanie, et associés le plus souvent aux génotypes VHB/E et VHB/A [120].

### Aspects diagnostiques

Les IgM anti-Delta sont les premières produites, concomitamment aux IgM anti-HBc dans le cas de co-infection. Elles sont détectables 2 à 4 semaines après une surinfection Delta. En cas d'infection résolutive, elles disparaissent en 12 semaines. À la différence des autres infections virales, les IgM peuvent persister dans l'hépatite Delta chronique. Leur présence indique une infection active. Cependant, elles peuvent être absentes chez certains patients. Ce marqueur n'est donc pas assez informatif pour le diagnostic ou le suivi des patients, tout comme le dosage de l'Ag Delta dont la présence, lors de l'infection aiguë, est fugace.

Les IgG anti-VHD produites chez les sujets ayant été infectés par le VHD persistent dans le sérum des patients, que l'infection aiguë ait été résolue ou qu'elle soit devenue chronique. Ainsi, la détection d'IgG anti-VHD est le test de dépistage essentiel affirmant un contact avec le VHD. Par contre il ne permet pas de conclure à une réplication active du virus.

L'ARN du VHD est détectable dans le sérum ou le plasma dès la 2^e^ semaine après l'infection. Sa présence signifie que l'infection est active, c'est donc le marqueur essentiel au diagnostic d'infection active par l'hépatite Delta.

En raison de la variabilité du génome du VHD et de la disparité des performances des trousses d'amplification génomique par PCR, des faux négatifs sont possibles. La mesure de la charge virale du VHD est essentielle en diagnostic et pour suivre l'efficacité des traitements antiviraux [15]. À cet effet, l'OMS a mis en place un standard international afin de permettre aux laboratoires d'exprimer leurs résultats en unités internationales (UI).

#### Accès au diagnostic en Afrique, tests rapides: un espoir

Le développement de méthodes diagnostiques alternatives pour le dépistage et le diagnostic de l'hépatite Delta est en plein essor. C'est le cas d'un nouveau test immunologique sur bandelettes permettant la mise en évidence des anticorps dirigés contre l'antigène Delta. Dévaluation des performances a montré une sensibilité et une spécificité excellentes par rapport à un test ELISA de référence. Il est compatible avec les matrices biologiques conventionnelles, et permet une biologie délocalisée auprès du patient ou « point of care testing ». Il sera donc d'un intérêt particulier vis-à-vis de populations sans accès aux structures de soins classiques, et particulièrement en Afrique [65].

### Aspects épidémiologiques

Les modes de contamination du VHD sont assez comparables à ceux du VHB, mais le virus de l'hépatite Delta se transmet essentiellement par voie horizontale (parentérale et sexuelle) [72]. En effet, dans la littérature la transmission mère-enfant (TME) semble exceptionnelle, probablement parce que le VHD inhibe la réplication du VHB, ce qui réduit ce risque [21].

La transmission horizontale survient dans l'enfance et au cours de l'adolescence en rapport avec le partage des objets de toilette ou tranchants; lors des tatouages ou des circoncisions; ou lors de gestes infirmiers, médicaux ou chirurgicaux (transmission nosocomiale) [72]. Ces modes de transmission semblent importants dans les régions à forte prévalence comme l'Afrique [20].

Les personnes toxicomanes par voie intraveineuse ont été particulièrement touchées, avec un risque d'infection par le VHD multiplié par 2,57. La prévalence du VHD chez les toxicomanes porteurs chroniques du VHB dans le monde se situe entre 14,57 et 37,57% selon les études [19].

Le VHD est aussi plus fréquent chez les personnes ayant des comportements sexuels à haut risque, les personnes ayant des partenaires sexuels multiples et des rapports sexuels non protégés ainsi que chez les hommes ayant des relations sexuelles avec les hommes (HSH) [19]. La prévalence élevée des Ac anti-VHD chez les PvVIH a aussi été confirmée par plusieurs études, comme nous le reverrons plus loin [30, 69, 83].

#### Répartition géographique mondiale

**Figure 2:**
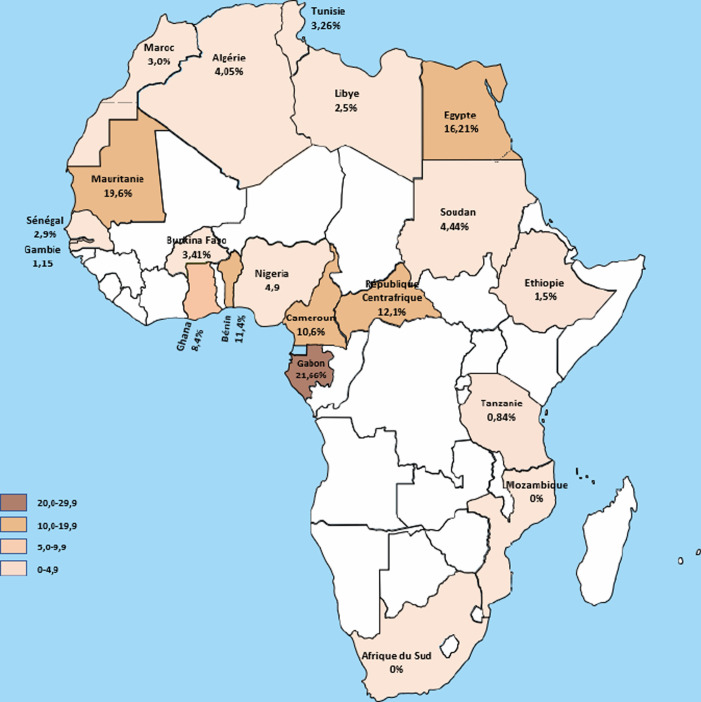
Prévalence de l'hépatite D en Afrique, d'après les publications référencées (lorsqu'il y avait plusieurs publications, nous avons fait la moyenne des études réalisées en population générale) [1, 10, 27, 41, 45, 51, 60, 76, 77, 100, 110] Prevalence of hepatitis D in Africa, after referenced publications (when there was more than one publication, we averaged the studies made in general population) [1, 10, 27, 41, 45, 51, 60, 76, 77, 100, 110]

La prévalence et la répartition génotypique du VHD varient considérablement. En raison d'un nombre limité d’études, la prévalence actuelle du VHD reste controversée. Une méta-analyse récente (2019) a révisé toutes les études publiées de 1997 à 2016 et la prévalence du VHD était évaluée à 0,98% en population générale et à 14,57% chez les porteurs chroniques du VHB [19]. Une autre étude de 2019 effectuée à partir d’études publiées entre 1980 et 2018 a suggéré un taux global de prévalence du VHD de l'ordre de 13,02%, ce qui correspond à une population comprise entre 48 et 60 millions d'individus dans le monde [82] (Fig. 2). Un autre travail, qui a analysé 282 études représentant un total de 120 293 sujets porteurs de l'AgHBs, a suggéré que la prévalence du VHD était d'environ 4,5% en 2020, soit environ 12 millions d'individus dont 7 millions de patients ayant une réplication virale [111].

L'infection par le VHD est donc répandue dans le monde entier, et sa prévalence ne suit pas celle du VHB [19].

#### Situation en Afrique

En Afrique subsaharienne, la prévalence des hépatites Delta est surtout élevée en Afrique de l'Ouest et en Afrique centrale [4]. Dans une revue systématique de 2017, la prévalence des anticorps anti-VHD chez les personnes infectées par le VHB variait de 26% en Afrique centrale, à 7% en Afrique de l'Ouest et de seulement 0,05% en Afrique orientale et australe [110]. Des zones de forte prévalence ont été signalées. Le Cameroun a enregistré une prévalence de 14%-35% en 2011 et le Nigeria de 5% en 2014 dans la population infectée par le VHB [110]. À Bangui, en République centrafricaine, une épidémie d'infection par le VHD aurait été à l'origine de cas d'hépatites aiguës, voire fulminantes, de pronostic sévère (88% de décès) dans les années 1980. La prévalence de l'infection par le VHD restait élevée en 2010 dans des populations asymptomatiques: étudiants (5,4%) et femmes enceintes (18,8%) [60].

En Afrique subsaharienne, la Mauritanie semble être un pays de prévalence exceptionnellement élevée. L'infection par le VHD toucherait 10 à 30% des patients infectés par le VHB (10 à 15% de la population) [74, 79]. Une enquête réalisée à l'hôpital, chez les patients suivis pour une infection par le VHB, a montré une prévalence de co-infection Delta de l'ordre de 30% avec, dans ce cas, des atteintes hépatiques plus sévères. Plus récemment, nous avons trouvé que plus de 60% des sujets cirrhotiques ou atteints de carcinome hépatocellulaire (CHC) étaient porteurs d'une co-infection VHB/VHD [118]. Dans la Figure 2, nous avons essayé d'actualiser, en 2023, les données de prévalence les plus récentes; lorsque plusieurs études étaient disponibles, nous avons fait une moyenne des prévalences rapportées.

### Aspects cliniques: histoire naturelle

#### Co-infection

Lors de la co-infection par le VHD et le VHB, les deux virus sont simultanément présents [11]. Les patients atteints d'une co-infection ont un risque élevé de maladie hépatique aiguë grave voire fulminante: environ 75% des personnes co-infectées ont des formes plus sévères qu'en cas de mono-infection par le VHB [38]. Après un délai d'incubation de 3 à 7 semaines, survient un épisode d'hépatite aiguë biphasique avec une réplication virale Delta importante.

L'hépatite Delta devient chronique dans seulement 10 à 30% en cas de co-infection et est cliniquement similaire à l'hépatite B chronique [72].

#### Surinfection

La surinfection survient lorsque le patient, déjà infecté par le VHB de façon chronique, est infecté dans un second temps par le VHD [44, 126]. Dans ce cas, il existe le plus souvent une diminution de la virémie du VHB [115].

L’épisode aigu survient 2 à 6 semaines après la contamination. Chez le patient dont on ignore le portage chronique de l'AgHBs, cet épisode peut être interprété à tort comme une hépatite aiguë B. Les formes fulminantes sont plus fréquentes, environ 15 à 20% des cas, que dans les hépatites virales B aiguës [59, 71]. Lorsque le portage de l'AgHBs est connu, la surinfection peut être interprétée comme un épisode de réactivation [59], c'est pourquoi on recommande toujours de réaliser une sérologie VHD lors d'une exacerbation d'une hépatite chronique B.

La surinfection par le VHD est caractérisée par un taux de passage à la chronicité de 60 à 90%. Les patients ont des formes chroniques sévères et une progression vers la cirrhose avec décompensation pouvant conduire au décès [59].

En effet, le risque de développer une cirrhose est trois fois plus élevé chez les patients infectés par le VHD par rapport à ceux atteints du VHB seul [2, 97]. La surinfection par le VHD est également associée à un risque multiplié par trois d’évolution vers le CHC et à un risque de mortalité doublé [96].

**Figure 3:**
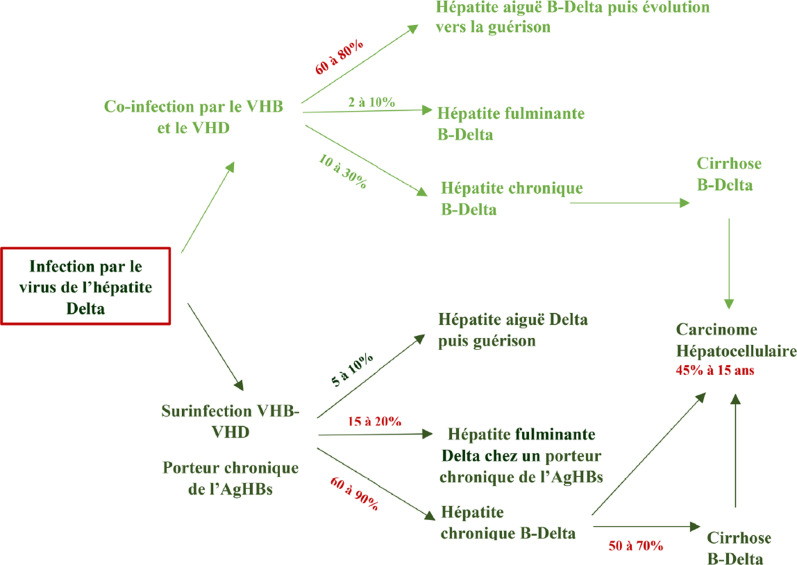
Histoire naturelle de l'infection à VHD Natural history of HDV infection

La Figure 3 résume les différentes phases de l'histoire naturelle de l'hépatite Delta. L'impact de l'infection à VHD sur l’évolution clinique de la cirrhose liée au VHB a été analysé dans plusieurs études [2, 97]. Dans l’étude française rétrospective du CNR de l'hépatite Delta réalisée chez 1112 patients, d’âge moyen de 36 ans, dont 58,7% de sujets originaires d'Afrique et 52,5% d'Afrique subsaharienne, 88% avaient un ARN VHD détectable et une infection par le génotype 1 du VHD dans 76% des cas. Environ 28% des patients étaient déjà au stade de cirrhose à l'inclusion et la moitié avaient déjà eu un épisode de décompensation. La cirrhose était moins fréquente chez les sujets d'origine africaine [97]. Une méta-analyse des études réalisées chez des patients atteints d'hépatite Delta comparés aux patients atteints d'hépatite B montre une augmentation globale du risque de CHC de 1,28, plus marquée dans les cohortes prospectives (2,77) et chez les PvVIH (7,13) [40].

L’évaluation de la sévérité de l'atteinte hépatique est, comme dans l'hépatite B, essentielle pour établir le stade de l'infection chronique et décider ou non de l'initiation d'un traitement. Les marqueurs non invasifs peuvent être utilisés. Ces tests ou méthodes n'ont cependant pas tous été validés chez les patients vivant avec l'hépatite Delta, en dehors du Fibroscan^®^ [25, 124]. Un autre test biologique, spécifique des hépatites Delta a récemment été développé, qui serait bien corrélé à l'atteinte histologique [24]. La PBH est cependant parfois nécessaire pour préciser le degré d'atteinte hépatique.

### Co-infection par le VIH et les virus des hépatites B et Delta

Les deux tiers des PvVIH vivent en Afrique subsaharienne [33], et plus de 100 millions de personnes sont porteuses chroniques du VHB en Afrique [85]. Selon les estimations actuelles, environ 7,6% (IC [5,6-12,1]) des personnes vivant avec le VIH (sur les 37,7 millions de PvVIH) ont une hépatite B chronique soit environ 2,7 millions de personnes [42, 90].

La prévalence de la co-infection VIH/VHB est variable suivant les pays [48]. Aux États-Unis et en Europe occidentale, l'infection chronique par le VHB est retrouvée chez 6 à 14% des PvVIH [40].

En Afrique, la prévalence de la co-infection VIH/VHB est très variable oscillant entre 10 et 20% selon les pays [40]. Elle a été estimée à 8% en République démocratique du Congo [58], 25% au Sénégal [30, 68], entre 12 et 17% au Burkina Faso [8], entre 9 et 23% en Côte d'Ivoire [6], entre 6,5 et 11,9% au Nigeria [50], 25% en Guinée-Bissau, 20% au Cameroun [98], 11% au Mozambique [102] et 8% en Afrique du Sud [22, 81]. En Mauritanie, une enquête réalisée en 2015 auprès de 100 patients VIH consécutifs a montré une prévalence de l'AgHBs de 27% et une prévalence des Ac anti-VHD de 41% (données non publiées).

L'immunosuppression liée au VIH modifie l'histoire naturelle du VHB et aggrave son pronostic [53, 57]. Le taux de passage à la chronicité est supérieur chez les PvVIH par rapport à la population générale: 20 à 90% *versus* 2 à 7% respectivement, en fonction des études [57, 73]. Le pourcentage de patients porteurs chroniques de l'AgHBs ayant un AgHBe positif est également plus élevé chez les patients co-infectés que chez les patients mono-infectés [67, 80].

Enfin et surtout, le VIH entraîne un risque plus élevé de progression de la fibrose et d’évolution vers la cirrhose et le CHC. L’âge, la charge virale VHB, le taux de lymphocytes T CD4 bas, la persistance de l'AgHBe et l'absence d'un traitement ARV incluant une molécule efficace contre le VHB, sont des facteurs de mauvais pronostic [18, 75].

### Co-infection VIH/VHB/VHD

#### Épidémiologie

Les données sur la prévalence du VHD chez les patients co-infectés par le VIH/VHB sont rares [90]. La prévalence du VHD varie selon la zone géographique [40]. En Europe de l'Ouest et en Amérique du Nord, la consommation de drogues injectables a favorisé l’émergence des infections par les virus VIH, VHB, VHC et VHD dans les années 1980 [94]. Actuellement, en Europe de l'Ouest, la plupart des nouveaux cas d'infection par le VHD chez les PvVIH sont identifiés chez des patients migrants [97].

Dans la cohorte prospective multicentrique EuroSIDA, une sérologie positive anti-VHD était retrouvée chez 14,5% des patients coinfectés VIH/VHB chroniques avec, pour la plupart, une virémie VHD détectable (86,8%). Une prévalence similaire de l'infection par le VHD a été observée pour la cohorte de PvVIH suisse SHCS (Swiss HIV cohort study).

En France, dans la cohorte multicentrique Epidemiological Study on hepatitis B Infection (EPIB) recrutée en milieu hospitalier, qui comportait une proportion forte de migrants, des marqueurs d'infection par le VHD ont été retrouvés chez 12,4% des PvVIH co-infectés par le VHB contre 5,9% des mono-infectés VHB en 2008, puis, en 2012, 9,7% et 5% respectivement [89].

Comme vu plus haut, l'Afrique est la zone de forte prévalence à la fois du VIH et des virus des hépatites B et Delta [23]. En Afrique de l'Ouest, une étude regroupant des cohortes VIH/VHB du Burkina Faso, de la Côte d'Ivoire et du Mali, rapportait une séroprévalence de 14,9% [23], de 25% en Guinée-Bissau [49], de 12% au Cameroun [10], de 72% au Nigeria [86], et de 3,5% au Ghana [7]. Ces chiffres doivent être interprétés avec prudence, vu la disparité des performances diagnostiques des tests utilisés pour la mise en évidence de l'infection par le VHD [55, 63, 70]. Une étude récente réalisée au Nigeria a analysé la prévalence des hépatites B et Delta chez 310 patients vivant avec le VIH dont 15 patients naïfs d'ARV. Les auteurs retrouvent une prévalence du VHB de 16,1%; 72% de ces patients avaient une charge virale VHB détectable. Parmi les seuls 50 patients infectés par le VHB, 8 (16%) avaient un ARN VHD détectable [86]. En Mauritanie, sur la cohorte de patients suivis au Centre de Traitement Ambulatoire de Nouakchott (environ 3000 par an), nous avons inclus 300 PvVIH consécutifs co-infectés VIH/VHB et nous avons trouvé une séroprévalence élevée (37%) de l'infection par le VHD, sachant que les patients co-infectés VIH/VHB représentent environ 17% des PvVIH suivis au CTA de Nouakchott. Cependant, la proportion de patients avec un ARN du VHD détectable était seulement de 41%, chiffre plus faible que dans nos études antérieures [78], réalisées chez les patients non porteurs du VIH (article soumis).

#### Évolution clinique de l'infection par le VHD dans les co-infections VIH/VHB

Plusieurs études ont été menées pour évaluer l'impact de l'infection par le VHD sur les personnes co-infectées par le VIH/VHB [9, 61, 103].

Dans l’étude EuroSIDA de 2011, un lien a été établi entre la positivité des Ac anti-VHD et un risque 4 fois plus élevé de décès par mortalité hépatique et 2 fois plus de mortalité toutes causes confondues, après un suivi médian de 7,5 ans [107]. De même, dans l’étude de la cohorte suisse sur le VIH de 2017, la coinfection par le VHD multipliait par 9 le risque de CHC et par 8 le risque de décès lié à des complications hépatiques avec un suivi médian de 8,7 ans [9].

En Afrique, aucune étude n'a cherché à évaluer les conséquences de la triple infection, en termes de sévérité de l'atteinte hépatique. Dans l’étude réalisée en Mauritanie (en cours de publication), nous avons analysé l’évolution de la fibrose chez les patients co-infectés ou non par le VHD, et nous montrons que l’évolution de la fibrose, évaluée par les tests sanguins, entre deux bilans (distants de 22 ± 8 mois) est significative, en particulier chez les patients triplement infectés. De surcroît, le degré d'aggravation de la fibrose (différence entre le premier et le dernier bilan) est significativement plus important chez les patients avec une charge virale Delta détectable, ce qui rejoint les données décrites sur l'histoire naturelle plus sévère de l'infection par le VHD chez les patients PvVIH [39, 123].

L'ensemble de ces données confirme donc que la co-infection par le VHD augmente le risque de complications hépatiques et la mortalité chez les patients co-infectés par le VIH/VHB, y compris en Afrique [28].

#### Perspectives thérapeutiques

La prise en charge de l'infection par le VHD n'avait que peu changé depuis plus de 30 ans et consistait en un traitement par l'interféron, avec le seul progrès de l'interféron pégylé [37]. Le développement de nouvelles thérapeutiques ciblant l'entrée du VHD dans l'hépatocyte, la prénylation et la réplication virale permettent désormais d'espérer un traitement plus efficace contre l'infection par le VHD [117].

L'OMS recommande les analogues nucléotidiques ou nucléosidiques – ténofovir (TDF) ou entécavir (ETC) – pour le traitement de l'infection chronique par le VHB; cependant, ils n'ont pas ou peu d'impact sur la réplication du VHD [13]. Ils seront plus particulièrement réservés aux patients, rares, qui ont un AgHBe positif et/ou une charge virale VHB détectable et supérieure à 2000 UI/L, et surtout en cas de fibrose avancée, quelle que soit la charge virale VHB.

L'interféron alpha pégylé (IFN PEG) était le seul médicament considéré comme efficace contre le VHD dans les recommandations de l'OMS [89]. Il est actuellement recommandé pendant 48 semaines. Cependant, même aujourd'hui, il reste peu disponible en Afrique et son utilisation nécessite une conservation à +4 °C, ce qui est parfois compliqué. Parmi les patients qui ont reçu de l'IFN PEG pendant 48 semaines, 17 à 43% des patients traités avaient un ARN VHD indétectable [46]. Cependant, la réponse virologique soutenue demeure rare, et des rechutes tardives sont observées chez plus de 50% des répondeurs [99]. Certains auteurs ont proposé des traitements plus longs, avec parfois des négativations de l'AgHBs et de l'ARN Delta associées à une amélioration des lésions hépatiques. Dans une étude où une cohorte de 12 patients a été traitée par Peg-IFNa-2a pendant une médiane de 6,1 ans (intervalle 0,8-14,3), 4 patients ont obtenu une réponse complète avec séroconversion de l'AgHBs [47].

Concernant les nouvelles thérapeutiques, le bulévirtide est un inhibiteur d'entrée des hépatocytes qui inhibe l'entrée du VHD [117] en se fixant de façon compétitive sur le récepteur de l'AgHBs, le NTCP (polypeptide cotransporteur du taurocholate de sodium). Il est administré en association avec le TDF pendant 24 semaines, et entraîne une baisse significative de l'ARN du VHD. Cependant, la charge virale VHD augmente à l'arrêt du bulévirtide. L'ajout de l'IFN PEG au bulévirtide augmente le taux de réponse [54].

Actuellement, il est administré le plus souvent en combinaison avec l'interféron pégylé pendant 48 semaines avec de meilleurs taux de réponse qu'avec l'IFN PEG seul [122, 128].

Il est commercialisé, sous forme injectable (AMM dans l'hépatite B et Delta) en France sous le nom d'Hepcludex. Son coût élevé limite son accès. Il n'est pas encore disponible actuellement en Afrique subsaharienne.

La seconde molécule d'intérêt récente est le lonafarnib. C'est un inhibiteur d'assemblage du VHD qui bloque la farnesyl transférase. Plusieurs essais en association avec le ritonavir et l'IFN PEG pendant 24 semaines semblent montrer une action synergique [127, 128]. Il a l'avantage d’être actif sous forme orale, mais n'est pas encore disponible.

Enfin, l'interféron lambda pégylé, qui a une meilleure tolérance que l'IFN PEG semble donner des résultats intéressants [125], en particulier en association avec le lonafarnib [29].

## Conclusion

L'infection par le VHD demeure une maladie plus que négligée dans les pays à ressources limitées comme l'Afrique. Sa prévalence est importante dans la plupart des pays d'Afrique subsaharienne ou de l'Ouest. Cependant, peu d’études ont été réalisées, en particulier concernant sa morbi-mortalité. On ne peut qu'encourager les études en population générale ainsi que les études sur la morbidité de l'hépatite Delta et particulièrement son impact sur la survenue du CHC, troisième cause mondiale de mortalité par cancer.

Enfin, la vaccination contre l'hépatite B, dès la naissance, est l'intervention qui permettra au mieux de réduire la prévalence des hépatites B et Delta. L'accès au traitement de référence de l'hépatite Delta, l'interféron alpha pégylé, doit être également facilité.

## Contribution des auteurs

Conceptualisation: FLF, AEB

Bibliographie: AEB, CTA

Supervision: HLGG FLF

Validation: HLGG FLF

Rédaction: AEB HLGG FLF

Révision: HLGG FLF

## Liens d'intérêts

Les auteurs déclarent n'avoir aucun conflit d'intérêts.
